# Microbiome of diseased and healthy implants—a comprehensive microbial data analysis

**DOI:** 10.3389/fcimb.2024.1445751

**Published:** 2024-08-29

**Authors:** Pingyi Jia, Xinran Guo, Jinchen Ye, Hongye Lu, Jingwen Yang, Jianxia Hou

**Affiliations:** ^1^ Department of the Fifth Division, Peking University School and Hospital of Stomatology and National Center for Stomatology and National Clinical Research Center for Oral Diseases and National Engineering Research Center of Oral Biomaterials and Digital Medical Devices and Beijing Key Laboratory of Digital Stomatology and National Healty Center (NHC) Key Laboratory of Digital Stomatology and National Medical Products Administration (NMPA) Key Laboratory for Dental Materials, Beijing, China; ^2^ Peking University School and Hospital of Stomatology and National Center for Stomatology and National Clinical Research Center for Oral Diseases and National Engineering Research Center of Oral Biomaterials and Digital Medical Devices and Beijing Key Laboratory of Digital Stomatology and National Healty Center (NHC) Key Laboratory of Digital Stomatology and National Medical Products Administration National (NMPA) Key Laboratory for Dental Material, Beijing, China; ^3^ Stomatology Hospital, School of Stomatology, Zhejiang University School of Medicine, Zhejiang Provincial Clinical Research Center for Oral Diseases, Key Laboratory of Oral Biomedical Research of Zhejiang Province, Cancer Center of Zhejiang University, Engineering Research Center of Oral Biomaterials and Devices of Zhejiang Province, Hangzhou, China; ^4^ Department of Prosthodontics, Peking University School and Hospital of Stomatology and National Center for Stomatology and National Clinical Research Center for Oral Diseases and National Engineering Research Center of Oral Biomaterials and Digital Medical Devices and Beijing Key Laboratory of Digital Stomatology and National Healty Center (NHC) Key Laboratory of Digital Stomatology and National Medical Products Administration National (NMPA) Key Laboratory for Dental Materials, Beijing, China; ^5^ Department of Periodontology, Peking University School and Hospital of Stomatology and National Center for Stomatology and National Clinical Research Center for Oral Diseases and National Engineering Research Center of Oral Biomaterials and Digital Medical Devices and Beijing Key Laboratory of Digital Stomatology and National Healty Center (NHC) Key Laboratory of Digital Stomatology and National Medical Products Administration National (NMPA) Key Laboratory for Dental Materials, Beijing, China

**Keywords:** oral microbiome, dental implants, peri-implantitis, 16S rRNA, data analysis

## Abstract

**Objective:**

The purpose of this systematic bioinformatics analysis was to describe the compositions and differences in submucosal microbial profiles of peri-implants’ diseases and healthy implant.

**Material and methods:**

PubMed, Embase, ETH Z, Scopus, CNKI, and Wanfang databases were searched to screen relevant literature on the analysis of peri-implant microflora based on the sequencing analysis technique of 16S ribosomal RNA (16S rRNA) gene. High-throughput sequencing of the 16S rRNA gene of microorganisms from healthy implants, peri-implant mucositis, and peri-implantitis was downloaded from the screened articles. EasyAmplicon and Usearch global algorithm were used to match the reads from each dataset to a full length of 16S rRNA or ITS gene sequence. The microorganisms based on the Human Oral Microbiome Database (HOMD) were re-classified, and the microbial diversity, flora composition, and differential species of the samples were re-analyzed, including taxonomic classification and alpha and beta diversity calculations. The co-occurrence network was also re-analyzed.

**Results:**

A total of seven articles with 240 implants were included. Among them, 51 were healthy implants (HI), 43 were in the peri-implant mucositis (PM) group, and 146 were in the peri-implantitis (PI) group. A total of 26,483 OTUs were obtained, and 877 microorganisms were annotated. The alpha diversity including Chao1 (healthy implants, 121.04 ± 92.76; peri-implant mucositis, 128.21 ± 66.77; peri-implantitis, 131.15 ± 84.69) and Shannon (healthy implants, 3.25 ± 0.65; peri-implant mucositis, 3.73 ± 0.61; peri-implantitis, 3.53 ± 0.67) of the samples from the three groups showed a significant difference. The beta diversity of the three samples was statistically different among groups. The genera of *Treponema* and *Fretibacterium* were significantly more abundant in the PI group than in the other two groups, and the genus of *Streptococcus* was more abundant in the HI group. The relative abundance of *Porphyromonas* in the peri-implantitis group was 6.1%. The results of the co-occurrence network showed differences in the network topology among the three groups of samples. The most connected three genera in the healthy implants were *Halomonas*, *Fusobacterium*, and *Fretibacterium*. The most connected three genera in peri-implant mucositis were *Alistipes*, *Clostridia UCG-014*, and *Candidatus Saccharimonas*. The most connected three genera in the peri-implantitis group were *Lachnoanaerobaculum*, *Fusobacterium*, and *Atopobium*. The betweenness of *Porphvromonas gingivalis* (red complex) in the PI group (7,900) was higher than in the HI group (23).

**Conclusions:**

The community compositions of peri-implant submucosal microorganisms were significantly different in healthy implants, peri-implant mucositis, and peri-implantitis. The submucosal microbial communities in peri-implantitis were characterized by high species richness and diversity compared with the healthy implants; the relative abundance of red complex, some members of the yellow complex, and some novel periodontal pathogens was higher in the peri-implantitis and peri-implant mucositis groups than in the healthy implant group. The core flora of the co-occurrence network of healthy implants, peri-implant mucositis, and peri-implantitis varied considerably. The peri-implantitis site presented a relative disequilibrium microbial community, and *Porphyromonas* may play an important role in the co-occurrence network.

## 1 Introduction

Peri-implantitis has been defined as a plaque-associated pathological condition affecting tissues around dental implants (Berglundh et al., 2018; Doornewaard et al., 2017). A systematic review of the incidence of peri-implantitis showed that, at the patient level, the average prevalence of peri-implantitis was 19.53%, and at the implant level, it was 12.53% (Diaz et al., 2022). Peri-implantitis can lead to progressive bone loss, making it the main cause for dental implant failure (Heitz-Mayfield et al., 2018). Numerous experimental and clinical studies have shown that peri-implantitis is a pathological condition related to plaque formation in the tissues surrounding dental implants (Berglundh et al., 2018; Darby, 2022). Studies, which were conducted by traditional microbial culture techniques, have found that there was a significant difference in the composition of plaque biofilm between infected implants and healthy implants (Mombelli and Mericske-Stern, 1990; Hannig, 1997). Imbalances in the oral microbiome and plaque accumulation are closely associated with the development of peri-implant mucositis and peri-implantitis. Early attempts to identify and classify the bacterial components of subgingival plaque from peri-implantitis relied on bacterial cultivation techniques, with a focus on known periodontal pathogenic bacterial species (Mombelli and Mericske-Stern, 1990; Shibli et al., 2008). However, approximately 53% of oral microorganisms have not yet been named, and 35% have not yet been cultured (Chen et al., 2010). Differences in cultivation environments can also lead to biases in microbial composition, missing other possible microbial communities associated with peri-implantitis. Therefore, traditional bacterial cultivation techniques have technological limitations when they were applied to identify the dental implant microbiome’s composition.

At present, sequence analysis of the 16S ribosomal RNA (rRNA) gene is a powerful mechanism to identify new pathogens in patients with suspected bacterial disease, and more recently this technology has been applied in the clinical laboratory for routine identification of bacterial isolates (Patel, 2001). Therefore, 16S rRNA sequencing has become the main method to study the composition and distribution of microbial communities. It has been widely applied in the study of oral microbial communities under non-culture conditions (Sun et al., 2023). The Human Oral Microbiome Database (HOMD) is an international standard human oral microbiome bioinformatics database, which covers more than 600 types of prokaryotic microorganisms in the oral cavity and is used for data sharing of oral microorganisms (Chen et al., 2010). Similarly, the Oral Microbiome Bank of China (OMBC) was established in 2018 to build a Chinese-related oral microbiome bioinformatics platform (Xian et al., 2018).

In recent years, it has been found that the submucosal flora around an implant is an independent ecosystem with its unique community structure (Belibasakis and Manoil, 2021). The uniqueness of the microbial community in peri-implantitis infections is well acknowledged compared with periodontal pathogens. Koyanagi T et al (Koyanagi et al., 2010). first studied the submucosal biofilm of dental implants using 16S rRNA gene clone library technique in 2010. Some specific groups of microorganisms, belonging to *Chloroflexi*, *Tenericutes*, *Synergistetes*, and *Firmicutes*, were only found in peri-implantitis lesions. Kumar PS et al (Kumar et al., 2012). used second-generation sequencing technology to compare the submucosal flora between implants and natural teeth, showing that the abundance of Gram-negative anaerobic bacteria in normal implant teeth was higher than that in peri-implantitis and periodontitis sites. Many literatures showed submucosal microbiomes similarity between per-implantitis and periodontitis sites and found the complexity and uniqueness of peri-implant-related bacterial communities (Yu et al., 2019; Kotsakis and Olmedo, 2021).

Recently, a few studies have used 16S rRNA sequencing to establish the core microbiota around an implant. Results vary from study to study. The amplification of the V1–V3 region of the 16S rRNA gene was performed in some studies (Zheng et al., 2015; Nie et al., 2020), while the amplification of the V3–V4 region of the 16S rRNA gene was performed in other studies (Maruyama et al., 2014; Komatsu et al., 2020; Yu et al., 2019). Due to the heterogeneity of the papers, it is impossible to analyze data from published articles in a straightforward aggregated manner. Recently, high-throughput sequencing of the full gene of 16S rRNA has become a widely accepted technique (Johnson et al., 2019). Sequencing the entire 16S rRNA gene provides real and significant advantages over sequencing a partial 16S rRNA gene. None of the variable regions covered by partial 16S sequencing were able to recapture the diversity represented when sequencing the full-length gene (Yarza et al., 2014). High-throughput sequencing of the full gene is more accurate but expensive. Research to explore core microbiota around an implant base on full-length 16S rRNA gene sequencing were rarely reported. Amplicon sequencing and Usearch global algorithm could be used to match the reads from a partial region to a full length of 16S rRNA (Nishio et al., 2023; Rognes et al., 2016; Yuan et al., 2020). With the help of these methods, data from literatures based on different gene regions could be integrated and re-analyzed. In this paper, the partial region gene reads from previous implant-related literatures based on various gene regions were matched to a full length and then re-classified and summarized based on a full length of 16S rRNA gene sequencing and HOMD. The microorganisms in healthy implants and their disease states were re-explored.

## 2 Materials and methods

### 2.1 Literature collection

**Figure 1:**
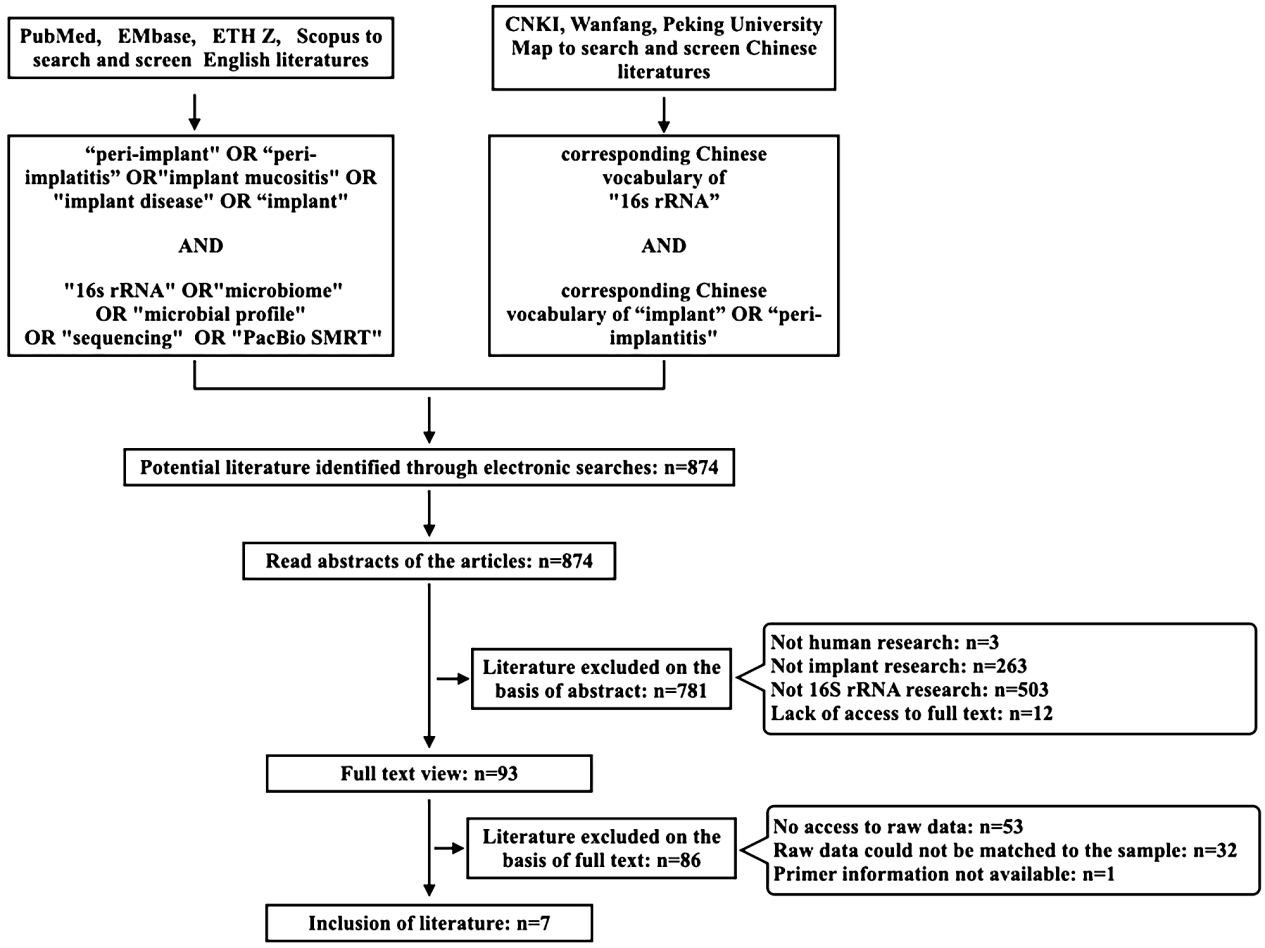
Potentially relevant available literature search flow.

Search terms identified using MeSH search terms relevant to the focus area were combined and applied using Boolean operators, “OR” or “AND”, as appropriate in the searched databases. The exact search term used was (“peri-implant” OR “peri-implantitis” OR “implant mucositis” OR “implant disease” OR “implant”) AND (“16s rRNA” OR “microbiome” OR “microbial profile” OR “sequencing” OR “PacBio SMRT”). Systematic searches were performed at the academic databases PubMed, EMbase, ETHZ, and Scopus. At the same time, corresponding Chinese vocabulary of “16s rRNA” AND “implant” OR “peri-implantitis” were used as keywords in the Chinese database CNKI, Wanfang, Peking University Map to search and screen Chinese literatures. Two co-investigators (Xinran Guo and Jinchen Ye) independently performed systematic searches of the aforementioned databases and sources. The search results were screened based on manuscript titles to select studies for abstract review, and then, based on the abstract reading, studies were identified for full-text analysis. Any disagreement among the co-investigators in the selection of studies for screening and/or for final inclusion was resolved by mutual discussion. Studies meeting the eligibility criteria were included for data extraction. All literatures published between 2010 and 2024 (up to July 2024) were reviewed and searched (**Figure 1**).

### 2.2 Inclusion criteria

Following are the inclusion criteria: (1) 16s rRNA gene-based Sanger sequencing (first-generation sequencing), high-throughput sequencing (second-generation sequencing), and PacBio SMRT (third-generation sequencing); (2) the research objects are humans; and (3) the diagnosis of the subject meets the criteria for “healthy implant”, “peri-implantitis”, and/or “peri-implant mucositis”, in accordance with previous research (Berglundh et al., 2018; Caton et al., 2018).

### 2.3 Exclusion criteria

The exclusion criteria are as follows: (1) the original literature was not available, (2) the original data and primer sequences of the experimental study were not available, and (3) the original data lacks relevant sample information.

### 2.4 Bioinformatics analysis and statistical analysis

The original sequencing data, downloaded from the screened articles, were divided into three groups, including healthy implant (HI), peri-implant mucositis implant (PM), and peri-implantitis implant (PI) based on the clinical diagnosis of the samples. Serial reading quality was checked by using the FastQC V. 0.11.5 program. The two ends of sequencing reads were processed by join with join _ -pair _ ends.py program in QIIME (version 1.9.0) and then trimmed to a Phred score of at least 20 by using split _ libraries _ fastq. py program in QIIME (version 1.9.0). The EasyAmplicon pipeline (https://github.com/YongxinLiu/EasyAmplicon) and Usearch global algorithm in VSEARCH 2.1.3 were then used to match the reads from each dataset to a full length of 16S rRNA. This process is based on a predefined set of sequences with known classifications (the manually curated Greengenes database 13.8 or the Unite database 7.2) (Rognes et al., 2016). The sequences are clustered into OTUs. The sequences of operational taxonomic units with 97% similarity were matched with the sequences in the Human Oral Microbiome Database (HOMD-V15.2). The class confidence was set to 0.7, and the data were sampled for species annotation analysis. The Chao1 index, richness index, Shannon index, and Simpson index were calculated at the OTU level to assess alpha diversity. Principal component analysis (based on Weighted UniFrac distance) was performed at the OTU level to assess beta diversity. An analysis of similarities (ANOSIM) was applied to compare the differences in flora structure among groups. Kruskal–Wallis test was performed to compare the microbial relative abundance at the phylum, genus, and species levels, respectively, and the false discovery rate (FDR) was calculated for the ***P***-value to reduce the false positive rate and to analyze the species with differences between groups. SPSS 26.0 software (IBM Corporation) and GraphPad Prism 9 were used for statistics and graphing. The difference was statistically significant when bilateral *P* < 0.05. Linear discriminant analysis (LDA) effect size (LEfSe) was used to compare the relative abundances of the dominant species among the three groups. The co-occurrence network was constructed based on the Spearman correlation between OTUs of each sample. The OTUs whose frequency is less than 0.6 in all samples are eliminated, and the Spearman correlation between the OTUs were computed with the “Microeco” package in the R language; a co-occurrence network was constructed based on the threshold of Spearman correlation between OTUs with ***R*** value greater than 0. 6 and *P*-value less than 0. 05. The co-occurrence network was visualized by using Gephi 0.10 software, and each dot in the network represented an OTU. The node size was the abundance of each OTU, and the nodes were colored according to the module.

## 3 Results

### 3.1 General information and clinical indicators of implants

Through the literature search from 2010 to 2023, 863 articles were selected, and seven articles with 240 implants were included through further data collation. Three of the studies were cohort studies, two of them were case–control studies, and two studies were cross-sectional studies. Agency for Healthcare Research and Quality (AHQR) and Newcastle-Ottawa Scale (NOS) were used to evaluate the quality of these studies (Berkman et al., 2004; Wells et al., 2009) (**Table 1**). There were 51 healthy implants (HI), 43 peri-implant mucositis (PM), and 146 peri-implantitis (PI). General information is shown in **Tables 2** and **3**.

**Table 1:** Quality evaluation of the studies.

Author, year	Type of study	AHQR score	NOS score	Quality of study
Yu et al., 2019	Cohort		7	Median
Komatsu et al., 2020	Case control		7	Median
Zheng et al., 2015	Case control	6		Median
Sun et al., 2023	Cross-sectional	7		Median
Maruyama et al., 2014	Cross-sectional		7	Median
Nie et al., 2020	Cohort		9	High
Wang et al., 2021	Cohort		8	High

**Table 2:** Basic information of literature data available for research and analysis.

Author, year	Sequencing section region	Number of HI	Number of PI	Number of PM	Region
Yu et al., 2019	V3-V4	18	18	/	China
Komatsu et al., 2020	V3-V4	/	23	/	Japan
Zheng et al., 2015	V1-V3	10	6	8	China
Sun et al., 2023	V3-V4	13	48	35	China
Maruyama et al., 2014	V3-V4	/	24	/	Japan
Nie et al., 2020	V1-V3	10	14	/	China
Wang et al., 2021	V3-V4	/	11	/	China

HI, healthy implants; PI, peri-implantitis implants; PM, peri-implant mucositis implants.

**Table 3:** History of periodontitis, definition of peri-implantitis, healthy implants, and peri-implant mucositis.

Author, year	History of periodontitis	Definition of peri-implantitis	Definition of healthy implants	Definition of peri-implant mucositis
Yu et al., 2019	Yes	PPD ≥5 mm, with the presence of BOP and radiographic evidence of bone loss	Probing depths ≤4 mm, being negative for BOP, with no radiographic evidence for bone loss	/
Komatsu et al., 2020	Yes	Having RBL ≥3 mm and/or probing depths ≥6 mm, with BOP and/or SUP	/	/
Zheng et al., 2015	Not mentioned	(i) PPDX5 mm, (ii) BoP, and (iii) vertical bone loss more than 1.8 mm after the first year in function In addition to bleeding on probing/suppuration, demonstrated probing pocket depth of ≥6 mm or attachment loss/bone loss of ≥2.5 mm	/	BoP (+) and probing pocket depth of ≥4 mm
Sun et al., 2023	Not mentioned	PPD was increased from baseline and radiographs showed bone loss in addition to the initial bone remodeling; implants without initial information: light bleeding and/or pus spillage, PPD ≥6 mm, radiographs show ≥3 mm of bone loss X-ray shows ≥3 mm of bone loss	No inflammation of the mucosa around the implant, as evidenced by pink color, no redness or swelling, no light bleeding or pus; PPD was not increased from baseline, and the radiographs showed no bone loss in addition to the initial bone remodeling	/
Maruyama et al., 2014	Yes	PPD ≥4 mm, BOP and/or pus-discharge presence, and concomitant radiographic bone loss presence	/	/
Nie et al., 2020	Not mentioned	Probing depths ≥4 mm, bleeding on probing with/without suppuration, and marginal bone loss ≥2 mm according to radiographs	Probing depths ≤3 mm and without visual signs of inflammation or marginal bone loss	/
Wang et al., 2021	Not mentioned	a) Obvious inflammatory symptoms around the implant, b) bone loss revealed by X-ray examination, c) possible hemorrhage and suppuration, d) at least one implant site with periodontal PD ≥6 mm, e) PLI around the implant ≥2 points, and f) visible bleeding around the implant after probing, with an SBI of ≥2 points	/	/

### 3.2 Composition of submucosal flora of implant

A total of 26,483 OTUs were obtained, and 877 microorganisms were annotated.

**Figure 2:**
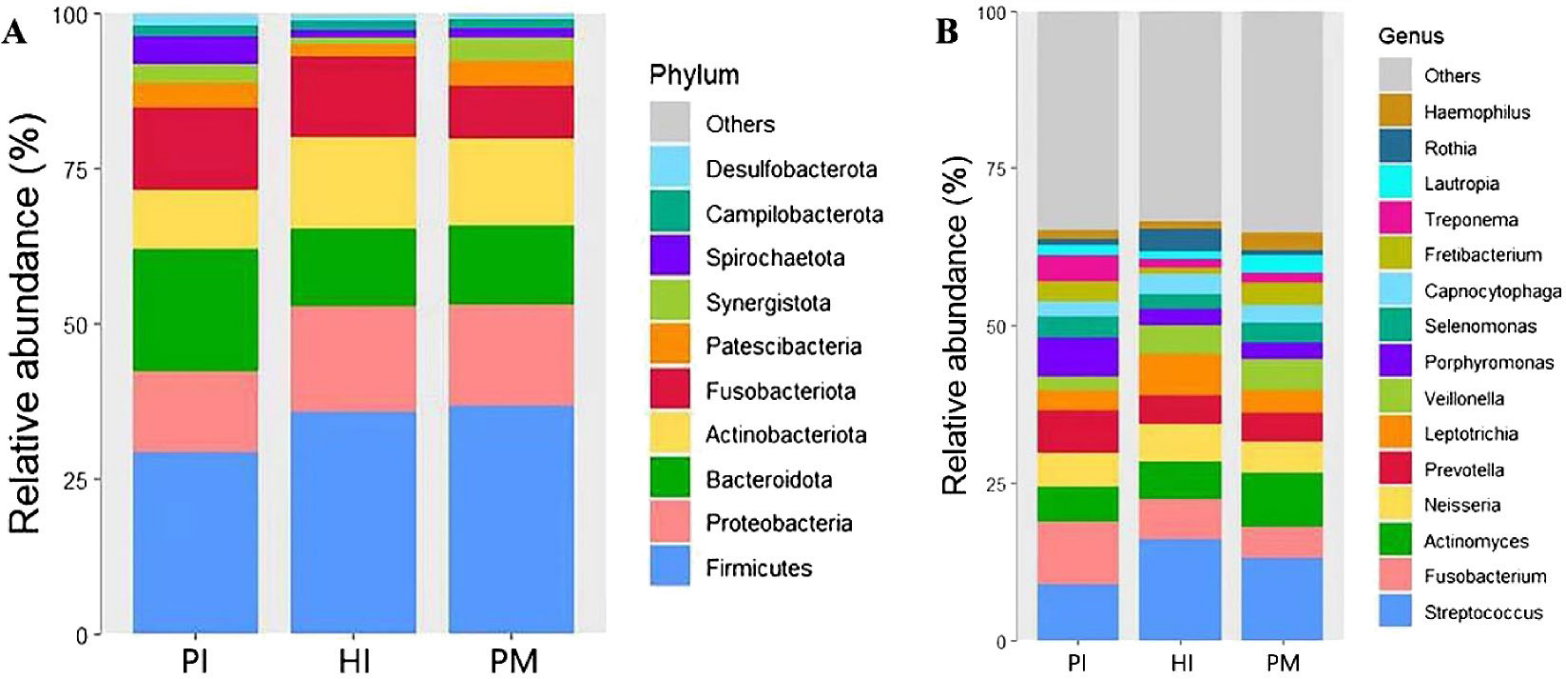
Species composition of submucosal microflora at the phylum and genus levels in healthy implants (HI), peri-implant mucositis (PM), and peri-implantitis (PI). **(A)** At the phylum level. **(B)** At the genus level.

At the phylum level (see **Figure 2A**), *Firmicutes*, *Proteobacteria*, *Bacteroidota*, *Actinobacteria*, *Fusobacteria*, and *Patescibacteria* were the predominant microflora with abundance >5% in healthy implants, peri-implant mucositis, and peri-implantitis, occupying more than 80% of all sequences. The remaining bacteria belong to *Synergistetes*, *Spirochaetes*, *Campilobacterota*, *Desulfobacteria*, and unclassified bacteria.

At the genus level (**Figure 2B**), a total of 15 dominant genera with abundance >2% were detected, including *Streptococcus*, *Fusobacterium*, *Actinomyces*, *Neisseria*, *Prevotella*, *Leptotrichia*, *Veillonella*, *Porphyromonas*, *Selenomonas*, *Capnocytophia*, *Fretibacterium*, *Treponema*, *Lautropia*, *Rothia*, and *Haemophilus*. Its total abundance accounts for more than 70% of the total sample sequencing.

**Figure 3:**
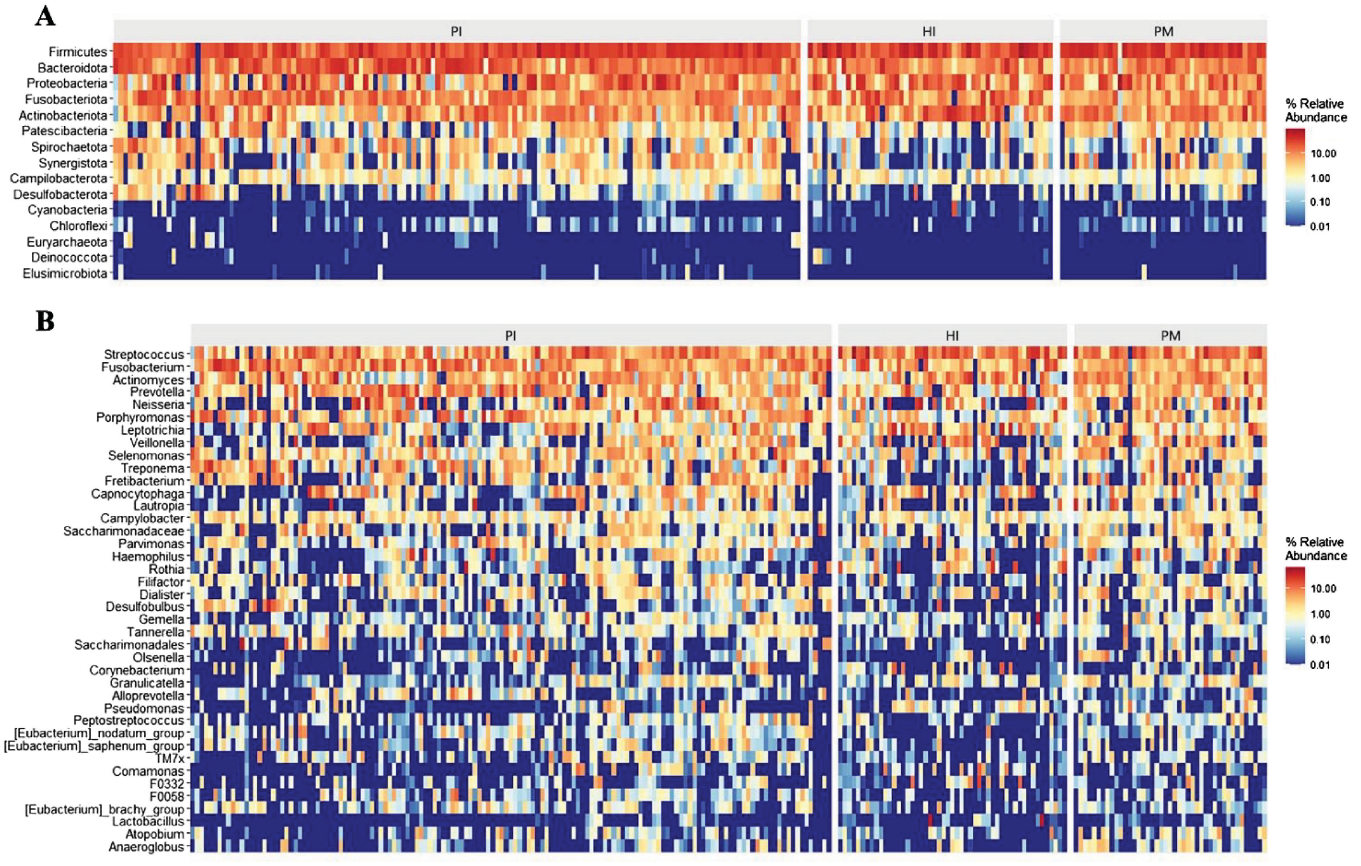
Microbial composition and abundance of submucosal microflora at phylum and genus levels in healthy implants (HI), peri-implant mucositis (PM), and peri-implantitis (PI). Heatmaps at the phylum level **(A)** and at the genus level **(B)**.

The heat map of species abundance in healthy implants, peri-implant mucositis, and peri-implantitis is shown at the phylum level in **Figure 3A** and at the genus level in **Figure 3B**. At the phylum level, the relative abundance of *Bacteroidota* and *Spirochaetota* in the PI group was significantly higher than the PM group (19.58% vs. 12.81%, *P* < 0.05; 4.34% vs.1.50%, *P* < 0.05). The relative abundance of *Fusobacteriota* in the PI group was higher (13.15%) than the PM groups (8.64%), but the difference was not statistically significant (*P* > 0.05). At the genus level, the relative abundance of *Treponema* in the PI group was significantly higher (4.32%) than the PM group (1.50%, *P* < 0.05). The relative abundance of *Porphyromonas* in the PI group was higher (6.10%) than the PM group (2.63%), but the difference was not statistically significant (*P* > 0.05).

### 3.3 Differences in the composition of submucosal flora of implants

**Figure 4:**
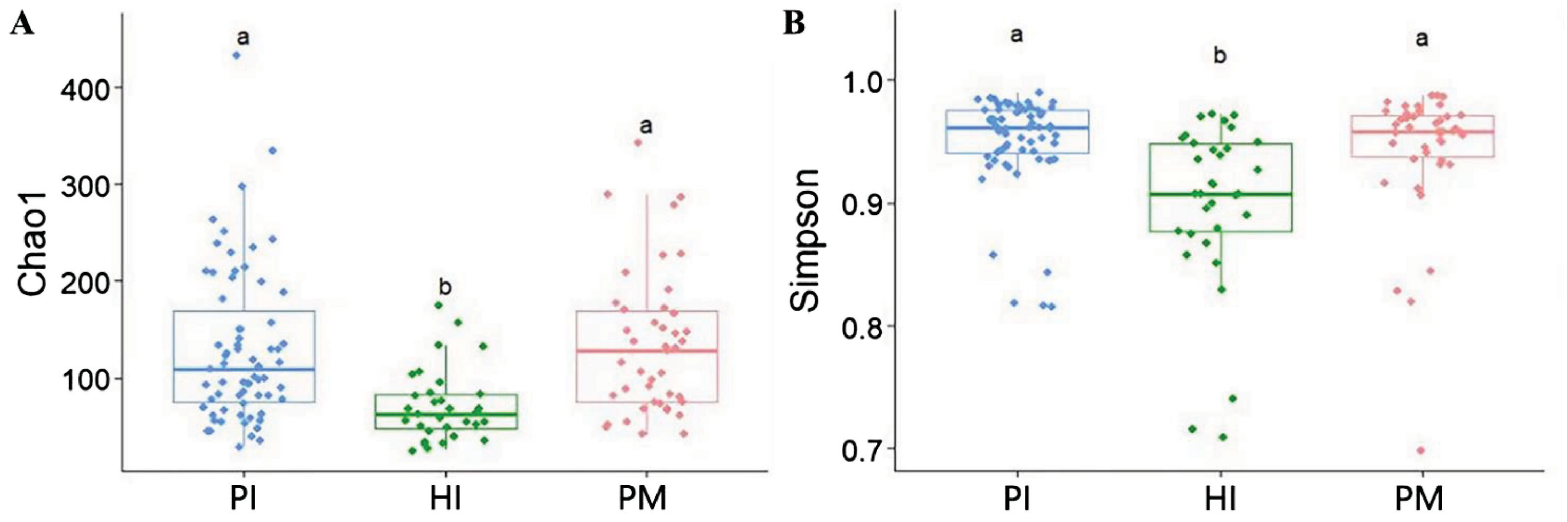
Comparison of α diversity of submucosal microflora of implants in peri-implantitis (PI), healthy implants (HI), and peri-implant mucositis (PM). **(A)** Microbial richness presented by Chao1 index. **(B)** Microbial diversity presented by Shannon index. Significant differences are marked with “a, b, c”; no common superscript denotes a significant difference (*P* < 0.05).

**Figure 5:**
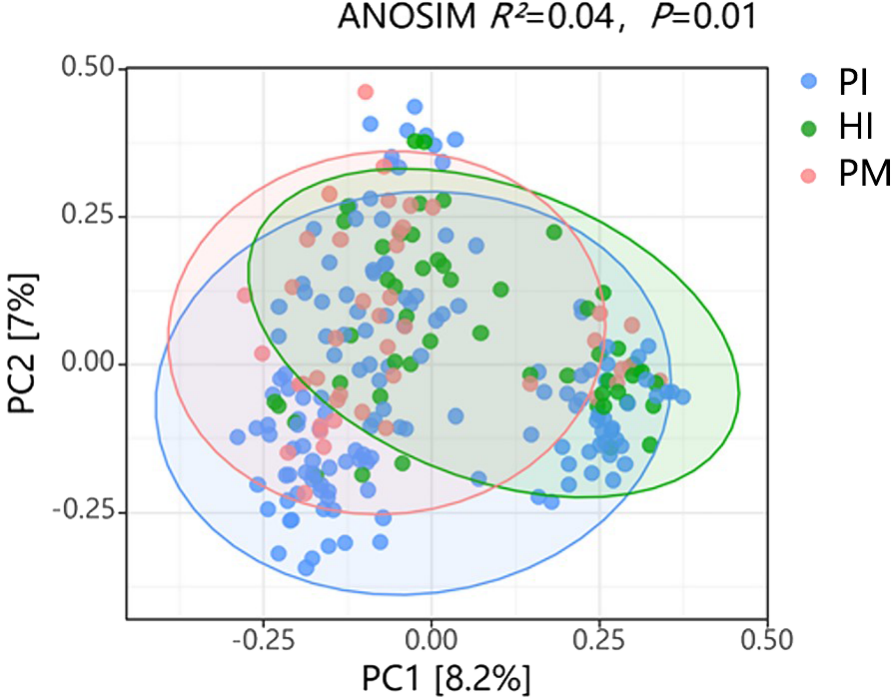
Comparison of β diversity of submucosal microflora in healthy implants (HI), peri-implantitis (PI), and peri-implant mucositis (PM). ANOSIM (analysis of similarities) *R* ^2^ = 0.04, *P* = 0.01. PC, principal component.

There was a significant difference in the alpha diversity of the submucosal flora between the implant disease group and the healthy group as indicated by the higher richness–Chaol index (healthy implants: 121.04 ± 92.76, peri-implant mucositis: 128.21 ± 66.77, peri-implantitis: 131.15 ± 84.69; **Figure 4A**) and diversity–Shannon index (healthy implants: 3.25 ± 0.65, peri-implant mucositis: 3.73 ± 0.61, peri-implantitis: 3.53 ± 0.67; **Figure 4B**). The results of the principal component analysis based on Weighted UniFrac distance showed that the species composition of submucosal flora in healthy implants, peri-implant mucositis, and peri-implantitis, respectively, were significantly different (beta diversity, *R* ^2^ = 0.04, *P* = 0.01, ANOSIM), and the principal component variables principal component 1 (PC 1) and PC2 were 8.2% and 7%, respectively (**Figure 5**).

**Figure 6:**
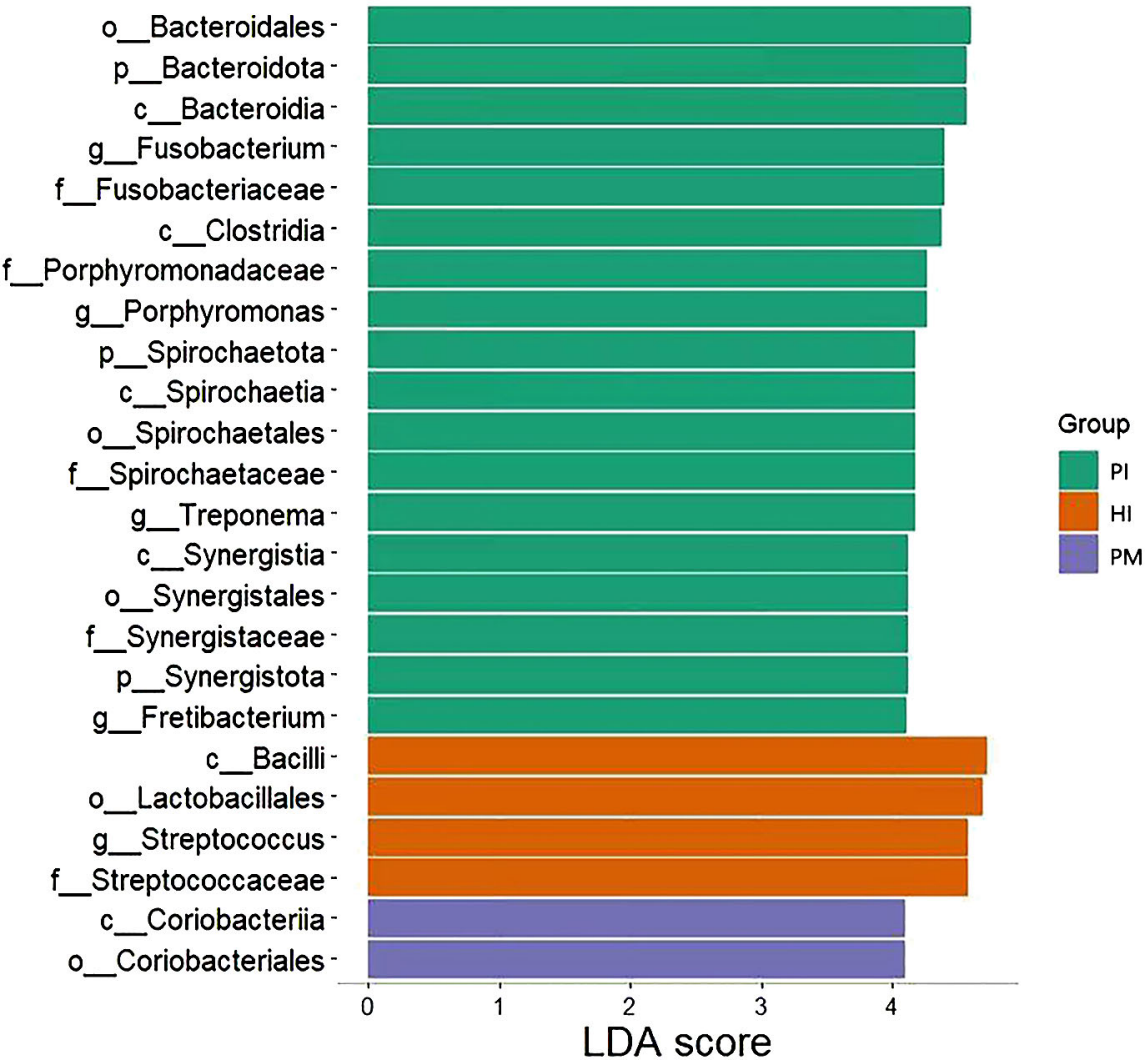
LEfSe analysis of submucosal flora of implants in healthy implants (HI), peri-implantitis (PI), and peri-implant mucositis (PM). LDA, linear discriminant analysis.

The analysis of the species composition of the submucosal flora in healthy implants, peri-implant mucositis, and peri-implantitis using LEfSe analysis is shown in **Figure 6**. Corrected differences between the two groups at different classification levels were screened with the aid of the Kruskal–Wallis test and the FDR-corrected *P*-values *P* < 0.05. At the phylum level, the abundance of *Bacteroidota* and *Spirochaetota* in the peri-implantitis group was significantly higher than that in the other two groups, and *Syntroph* was more abundant in the healthy implant group. At the class level, the abundance of *Bacteroidia* and *Clostridia* in the peri-implantitis group was significantly higher than that in the other two groups. *Bacillus* was more abundant in the healthy implant group than in the control group. There was a significant increase in *Coriobacteriia* in the peri-implant mucositis group. At the family level, the abundance of *Fusobacteriaceae*, *Porphyromonadaceae*, and *Spirochaetaceae* in the peri-implant group was significantly higher than that in other groups. The abundance of *Streptococcus* was higher in the healthy implant group. At the order level, the abundance of *Synergistales* in the peri-implantitis group was significantly higher than that in the other two groups. The abundance of *Lactobacillales* was higher in the healthy implant group, and *Coriobacteriales* was significantly increased in peri-implant mucositis. At the genus level, *Treponema* and *Fretibacterium* were significantly more abundant in the peri-implantitis group than in the other two groups, and *Streptococcus* was more abundant in the healthy implant group.

### 3.4 Co-occurrence network analysis reveals distinct microbial interaction patterns in healthy implant and peri-implant disease

**Figure 7:**
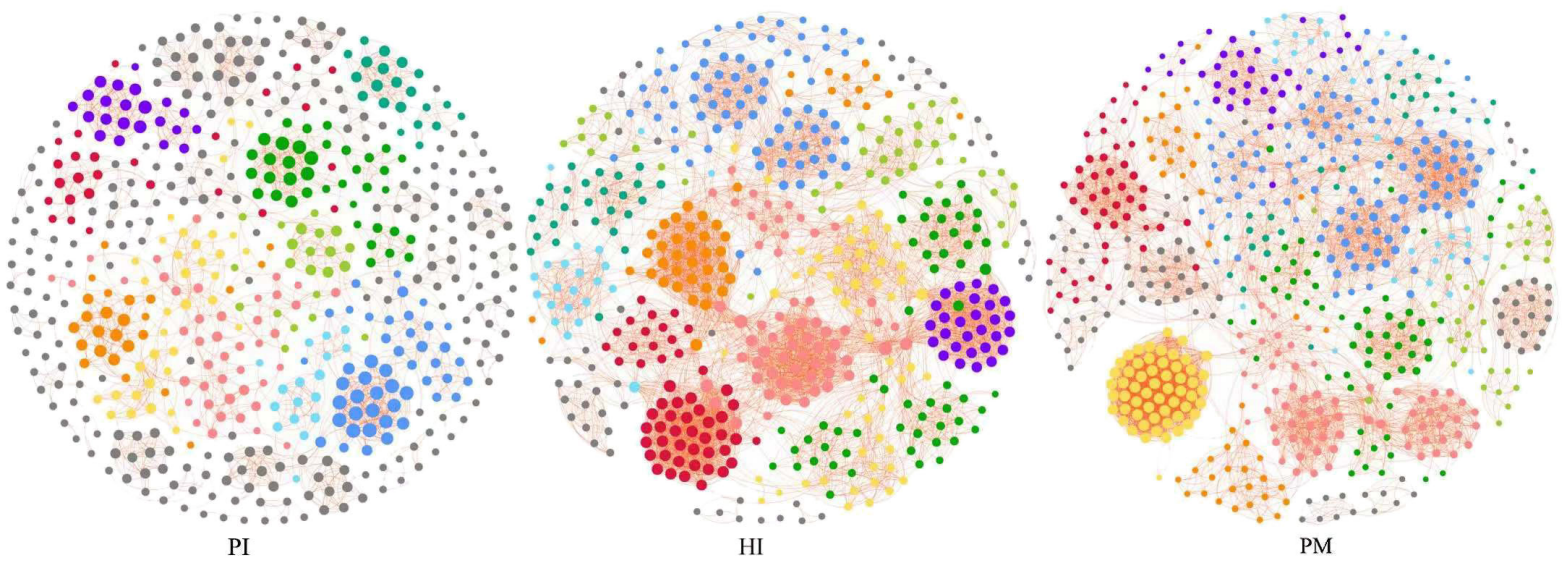
Co-occurrence network analysis: symbiotic network of three groups of microbial communities (network modules are shown in different colors). HI, healthy implants; PI, peri-implantitis; PM, peri-implant mucositis.

In this study, the method of co-occurrence network analysis was used to explore the potential relationship between the microbial communities under healthy implants, peri-implant mucositis, and peri-implantitis (**Figure 7**). The results show that there are differences in the network topology of the three groups. The connectivity, interaction, node number, and density of bacteria in the healthy implant group and the peri-implant mucositis group were more than those in the peri-implantitis group. The microbial community of the peri-implantitis sites was less complex compared with the peri-implant mucositis sites. Positive correlations between species were found in the three groups (**Supplementary Tables S1**-**S3**).

**Figure 8:**
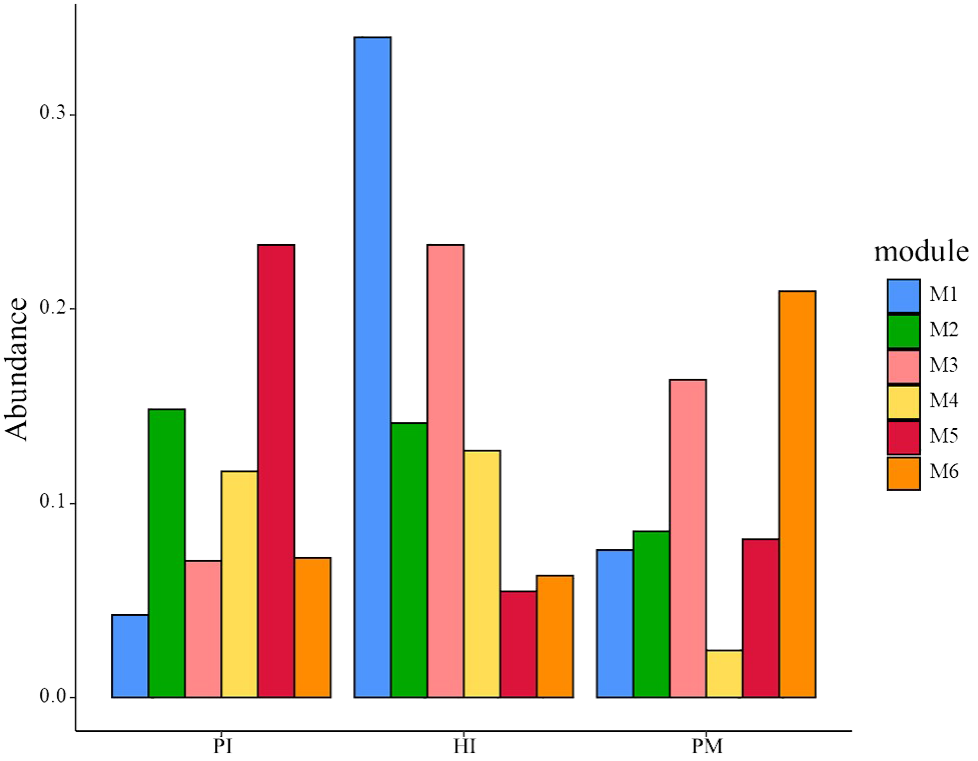
Relative abundances of the top six modules in three groups. HI, healthy implants; PI, peri-implantitis; PM, peri-implant mucositis.

The relative abundances of the top six modules of each group are shown in **Figure 8**. The highest relative abundance module in the HI group was module 1, including *Campylobacter_concisus* (green complex), *sputigena*, *Prevotella_saccharolytica*, etc. The highest relative abundance module in the PI group was module 5, including *Treponema_medium*, *Prevotella_intermedia* (oragen complex), etc. The highest relative abundance module in the PM group was module 6, including *Johnsonella_ignava*, *Actinomyces_israelii*, *Cardiobacterium_hominis*, etc. Interestingly, despite the fact that the relative abundance of module 4 in the PM group was not very high, the degrees of species were more than 40. Module 4 included *Erysipelotrichaceae_UCG-006*, *Peptostreptococcus_stomatis*, *Alistipes*, etc.

We found that the core bacteria of the three groups are quite different, and these core bacteria are the key nodes of the group’s co-occurrence network. The most connected three genera of healthy implants were *Fretibacterium* (degree 57, betweenness 6001), *Halomonas* (degree 55, betweenness 8246) and *Allorhizobium–Neorhizobium–Pararhizobium–Rhizobium* (degree 51, betweenness 1707). The most connected three genera in peri-implant mucositis were *Alistipes* (degree 56, betweenness 5094), *Clostridia UCG-014* (degree 55, betweenness 420), and *Candidatus Saccharimonas* (degree 55, betweenness 32). The most connected three genera in the peri-implantitis group were *Lachnoanaerobaculum* (degree 21, betweenness 3949), *Fusobacterium* (degree 21, betweenness 246), and *Atopobium* (degree 21, betweenness12). The betweenness of *Bacteroides forsythus* (red complex) in the PI group (9062) was higher than in the HI group (731). The betweenness of *Porphvromonas gingivalis* (red complex) in the PI group (7,900) was higher than in the HI group (23).

## 4 Discussion

Data from previous studies focusing on peri-implant microbial flora analysis based on 16s rRNA gene sequencing analysis technology was screened and downloaded in this study. The original sequencing data, downloaded from the screened articles, were divided into three groups, including healthy implant (HI), peri-implant mucositis implant (PM), and peri-implantitis implant (PI). Since two studies were conducted on the V1–V3 region and another five studies were conducted on the V3–V4 region, the Usearch global algorithm in VSEARCH 2.1.3 was then used to match the reads from each dataset to a full length of 16S rRNA or ITS gene sequence. The underlying database referred to (HOMD-V15.2) is also composed of full-length sequences. Then, the high-throughput sequencing samples of 16s rRNA gene of submucosal microorganisms in healthy implants, peri-implant mucositis, and peri-implantitis were analyzed. The microorganisms based on the Human Oral Microbiome Database (HOMD) were re-classified, and the microbial diversity, flora composition, and differential species of the samples were re-analyzed, including taxonomic classification and alpha and beta diversity calculations. The co-occurrence network was also re-analyzed. A total of 877 microorganisms were annotated.

Our results show that the dominant phyla of peri-implantitis are *Firmicutes* (29.13%), *Bacteroidota* (19.58%), *Fusobacteriota* (13.15%), and *Proteobacteria* (13.08%). The dominant phyla identified in this study in response to peri-implantitis was similar with the study of Sanz-Martin et al. (2017). Compared with the healthy implant, the abundance of *Firmicutes* was decreased and the abundance of *Bacteroidetes* was significantly increased in the peri-implantitis group. Similarly, a significantly higher relative abundance of *Bacteroidota* and *Fusobacterium nucleatum*, respectively, was found in the peri-implantitis group compared with healthy implant by Al-Ahmada et al. (*P* < 0.05). The major phyla of healthy implants were *Firmicutes* (51.9%), *Bacteroidota* (18.5%), *Clostridia* (11.1%), and *Proteobacteria* (7.4%), whereas the predominant phyla of peri-implantitis were *Firmicutes* (30.6%), *Bacteroidota* (40.3%), *Clostridia* (13.9%), and *Proteobacteria* (5.6%) (Al-Ahmad et al., 2018). Data were not included in this study because their data were not publicly shared. The dominant phylum around the implant changed according to different physiological conditions.

This study shows that the dominant genus of peri-implantitis are *Fusobacterium* (9.93%), *Streptococcus* (8.81%), *Prevotella* (6.79%), and *Prophyromonas* (6.10%). At the genus level, *Porphyromonas* and *Treponema* (4.33%), which are closely related to periodontal disease, were identified around peri-implantitis. However, the relative abundances of these periodontitis-related bacteria were lower in peri-implantitis sites than in periodontitis sites. This was consistent with the results of many previous studies (Kumar et al., 2012; Socransky et al., 1998; da Silva et al., 2014). Notably, the Human Oral Microbiome Database (HOMD) collected bacterial species distributed in the oral cavity region and the upper respiratory tract and found that the dominant phyla were *Actinobacteria*, *Firmicutes*, and *Proteobacteria* (Escapa et al., 2018). According to these studies, the flora distribution between periodontitis and peri-implantitis at the phylum level was different, and there were differences in the occurrence and development mechanism between peri-implant disease and periodontal disease. Similar results were also found in a previous study (Koyanagi et al., 2010).

Overall, microbiota had similar alpha diversity between the peri-implant mucositis (PM) and peri-implantitis (PI) groups. There were significant differences between them and the healthy implant group. The findings are consistent with previous studies (Hashimoto et al., 2022; Padial-Molina et al., 2024). Similar to a previous study (Li et al., 2015), the beta diversity of submucosal flora in healthy implants, peri-implantitis, and peri-implantitis was significantly different.

The abundance of *Fretibacterium* was higher in the peri-implantitis group than in the other two groups (**Figure 6**), which means it was one of the biomarkers of peri-implantitis. The effect of *Fretibacterium* on the development of periodontitis has been reported in the literature. This study suggests that this bacterium is related to periodontal pocket probing depth (PPD) and bleeding on probing (BOP) and positively correlated (*P* < 0.05) (Khemwong et al., 2019). Oliveira R et al. also reported related results, which proved that periodontitis is closely related to *Fretibacterium* (Oliveira et al., 2016). However, it has not been reported that this bacterium is significantly related to the occurrence of peri-implantitis. We have detected that there is a high species abundance in peri-implantitis by statistical analysis. Considering that it is an anaerobic *Bacillus*, we believe that its role and mechanism in the occurrence and development of peri-implantitis may be similar to those of periodontitis. However, its interaction with peri-implantitis needs further study.

In this study, we constructed a co-occurrence network of microorganisms based on Spearman’s rank correlation coefficients (Spearman coefficient >0.6). The connectivities between species in the three groups were quite different. The co-occurrence network of the peri-implantitis group was dominated by the red complex and showed a relative disequilibrium. Nie J et al. also found that the topology of the co-occurrence network of healthy implants is significantly different from that of peri-implantitis implants (Nie et al., 2020). It is well acknowledged that peri-implantitis is a multifactorial, dysbiosis-related pathological process (Kroger et al., 2018). Yu XL et al. found that there was a wide positive correlation between the bacteria of the synthetic bacteria phylum, including *Fusarium Acremonium*, *HMT 361/362*, and *Thiovibrio desthiolatus TG5* sp. in periodontitis and the peri-implantitis group. However, there was also a negative correlation in some genera, such as *Rosella*, *Microporus*, *Actinomyces*, *Corynebacterium*, *Streptococcus*, *Neisseria*, *Kingella*, *Leptotrichum*, *Fusobacterium*, etc (Yu et al., 2019). The three core bacteria in the peri-implantitis group in this study were *Lachnoanaerobaculum*, *Fusobacterium*, and *Atopobium.* Among them, *Lachnoanaerobaculum* has the highest connectivity in network co-occurrence. *Lachnoanaerobaculum* is obligate anaerobic, gram-positive (Ida et al., 2022), and it is reported as significantly increased in smokers’ subgingival plaque around an implant (Duan et al., 2017)*. Fusobacterium*, another core bacteria, was reported to play an important role in biofilm formation (Jiang et al., 2021) and could be distinctly detected in high-risk individuals (Wang et al., 2021). *Atopobium* was reported as over-represented in peri-implantitis sites compared with that in healthy sites (Barbagallo et al., 2022; Rubino et al., 2021). Large degrees of species including *Erysipelotrichaceae_UCG-006*, *Peptostreptococcus_stomatis*, and *Alistipes* were found in the peri-implant mucositis group. *Erysipelotrichaceae_UCG-006* was not yet reported in the dental field. *Peptostreptococcus stomatis* was significantly increased in the tumor sites of oral squamous cell carcinoma patients than in normal tissues (Luo et al., 2023). *Alistipes* was predicted to play a causal role in enhancing the risk of periodontitis (Zhang et al., 2019). Whether these organisms play an important role in the development of peri-implant mucositis needs to be further explored in the future.

Peri-implant mucositis is considered to be the precursor to peri-implantitis, a condition which may progress rapidly, leading to advanced bone loss and resulting in loss of an implant (Heitz-Mayfield, 2024). In this study, peri-implantitis sites harbor more anaerobic and hemophilic bacteria (*Fusobacteriota*, *Porphyromonas*, *Treponema*, and *Spirochaetota*) than sites with peri-implant mucositis. The microbial communities of peri-implantitis sites showed more imbalance compared with peri-implant mucositis sites with lower biomass. The abundance of *Coriobacteriia* and *Coriobacteriales*, respectively, was higher in the peri-implant mucositis group than in the other two groups (**Figure 6**). *Coriobacteriales* had a protective effect on allergic rhinitis and microscopic colitis (Jin et al., 2023; Sandler et al., 2023). Whether *Coriobacteriales* could impede the development of peri-implantitis from peri-implant mucositis sites needs further studies.

It should be noted that this study has limitations. Studies have shown that individual differences have a significant impact on the composition of the microbiota composition, which is caused by differences in the host immune response (Yu et al., 2019; Alves et al., 2022; Kensara et al., 2021). The literature data we collected were all from Asian populations, mainly Chinese and Japanese peri-implant samples. These individual selection biases may lead to a certain bias in the research results. To eliminate this bias, data collected from research based on other races is advocated.

To sum up, this study was conducted based on 16S rRNA sequencing literature data on submucosal microflora in healthy implants, peri-implant mucositis, and peri-implantitis. The composition and differences are re-analyzed comprehensively. The results showed that there was a significant difference in flora diversity between peri-implant disease and healthy implants. Some members of the red complex (*Fusobacterium* and *Prophyromonas*) and yellow complex (*Streptococcus*) are closely related to peri-implantitis. The abundance of *Fretibacterium* (an anaerobic *Bacilli*) was higher in the peri-implantitis group than in the other two groups. The co-occurrence network in the peri-implantitis group was different from that in the healthy implant group and the peri-implant mucositis group. The peri-implantitis site presented a relative disequilibrium microbial community, and members of the red complex (*Porphyromonas* and *Bacteroides forsythus*) played an important role in the co-occurrence network.

## Data Availability

Publicly available datasets were analyzed in this study. This data can be found here: NCBI Short Reads Achieve under the accession of biosamples: SAMN09464023 - SAMN09464094; NCBI Short Reads Achieve: PRJNA861252; Short Reads Archive (Accession number SRP043555); the DNA Data Bank of Japan (DDBJ) under accession no. DRA000946 (http://www.ddbj.nig.ac.jp/); the DNA Data Bank of Japan (DDBJ) with the following accession numbers: 16S rDNA sequencing (DRA010104) and metagenomic analysis (DRA006832); NCBI PRJNA786326; Sequence Read Archive with the accession number PRJNA487121.

## Supplementary material

Supplementary Table 1 Genera in co-abundance group used for network construction of peri-implantitis group.

Supplementary Table 2 Genera in co-abundance group used for network construction of healthy group.

Supplementary Table 3 Genera in co-abundance group used for network construction of peri-implant mucositis group.
